# Editorial: RNA recognition landscapes and anticancer drug targeting

**DOI:** 10.3389/fonc.2023.1222883

**Published:** 2023-06-19

**Authors:** Erica Salvati, Anna Lewinska, Erik Dassi, Maciej Wnuk, Vito G. D’Agostino

**Affiliations:** ^1^ Institute of Molecular Biology and Pathology, National Research Council, Rome, Italy; ^2^ Institute of Biotechnology, College of Natural Sciences, University of Rzeszow, Rzeszow, Poland; ^3^ Laboratory of RNA Regulatory Networks, Department of Cellular, Computational and Integrative Biology (CIBIO), University of Trento, Trento, Italy; ^4^ Laboratory of Biotechnology and Nanomedicine, Department of Cellular, Computational and Integrative Biology (CIBIO), University of Trento, Trento, Italy

**Keywords:** RNA, RNA-binding proteins, post-transcriptional control, drug, mechanism of action (MOA), anti-cancer, anti-inflammatory

The interplay between RNA-binding proteins (RBPs) and coding/non-coding RNAs creates an intricate network of connections that significantly impact cell physiology with consequences in cancer (1–3). Integrative proteo-transcriptomic analyses indicate the existence of dynamic RBP-RNA interactions only partially deciphered and involved in immunomodulatory effects (4), cell signaling (5), viability (6), metabolic reprogramming (7, 8), or secretory pathways (9). The relevance of these interactions also emerges when better investigating the mechanism of action of drugs with anti-inflammatory/anti-cancer activities, remarkably including the post-transcriptional regulation to exert their pleiotropic effects (10–13). Deciphering and prioritizing these interaction dynamics offer a tremendous opportunity to interfere with new mechanisms that could lead to novel synergistic, durable treatments for cancer.

In this Research Topic, we collected five articles as examples describing the post-transcriptional activity of a natural compound, the pathological functions of selected miRNAs and their interplay with long non-coding RNAs (lncRNAs), and the emerging prognostic significance of selected RBPs and lncRNAs in solid tumors.

Chen et al. characterized the Glaucocalyxin A (GLA)-induced anti-proliferative activities on ovarian cancer cells. This natural compound, deriving from *Rabdosia japonica*, was already known for anti-cancer and anti-inflammatory properties (14). The authors reported drug-induced cell phenotypes associated with high-mobility group box 3 (HMGB3) downregulation. They found that GLA concomitantly induces the expression of miR-374b-5p, matching complementarity with a specific region on HMGB3 3’UTR, significantly reducing HMGB3 protein expression and identifying a functional axis sustained by HMGB3 mRNA and miR-374b-5p interaction. This report adds new details about the mechanism of action of GLA, including a relevant post-transcriptional control strongly associated with the drug’s anti-cancer effects.

Lin et al. addressed soft-tissue sarcoma (STS). Although representing less than 1% of all human cancers, STS severely impacts cancer mortality due to the absence of therapies to tackle advanced disease stages. The authors interrogated The Cancer Genome Atlas (TCGA) and Genotype-Tissue Expression (GTEx) databases and compared STS with healthy tissues, identifying more than three hundred differentially expressed RBPs. Notably, nine out of those were found to predict patients’ 1-year, 3-year, and 5-year survival rates. These data could open new mechanistic studies on critical RBPs that could represent new therapeutic targets and/or improve the diagnosis and stratification of patients with soft-tissue sarcoma.

In the research article by Yuan et al., selected lncRNAs are presented as mediators of cuproptosis in hepatocellular carcinoma (HCC). With a similar approach using TCGA dataset on liver hepatocellular carcinoma and comparing normal *versus* tumor samples, the authors identified a signature of differentially expressed copper death-related lncRNAs with potential prognostic significance. Among ten copper death- and 22 copper metabolism-related targets, four lncRNAs, namely AL590705.3, LINC02870, KDM4A-AS1, and MKLN1-AS were validated using *in vitro* HCC cellular models supporting post-transcriptional mechanisms in cuproptosis pathways.

Li et al. identified an interplay between the lncRNA LBX2-AS1 and the microRNA mir-597-3p in osteosarcoma. The authors found this lncRNA as one of five targets composing a signature predicting tumor survival. Given its indicated role as an oncogene in several tumor types, the authors investigated the effects of LBX2-AS1 knockdown in osteosarcoma, detecting an increase in apoptosis and less severe phenotypes *in vivo*. By exploring potential miRNA targets as regulators of LBX2-AS1, the authors identified mir-597-3p as a potential sponged molecule, pointing out BRD4 as a target of this microRNA. Notably, they found an implication of the LBX2-AS1/mir-597-3p/BRD4 axis in the resistance to the JQ-1 BET bromodomain inhibitor, reporting that LBX2-AS1 silencing shifts the JQ-1 IC_50_ in osteosarcoma cells.

In the published review, Budi et al. summarize the latest knowledge on the roles of miR-128 in multiple cancer types. In particular, the authors discuss the physiological and pathological functions of miR-128, its biogenesis and molecular targets. The review contains details on the role of miR-128 in multiple malignancies such as breast cancer, lung cancer, thyroid cancer, head and neck cancer, osteosarcoma, glioma, leukemia, and multiple myeloma. The authors also discuss the role of miR-128 in the development of chemoresistance with implications for immunotherapy, offering a point of view on mechanisms underlying the progression and drug resistance of cancer cells potentially associated with a dysregulation of miR-128.

Several RNA-binding proteins and domains show preferred or flexible affinity towards a specific RNA consensus. The fate of recognized coding or non-coding RNAs depends on the competition between different RBPs that can “read the multiple codes” carried by single RNA substrates, forming dynamic post-transcriptional networks. This issue emphasizes the need to quantitatively unravel competitive interactions of RBPs and RNAs, including the characterization of RNA-binding sites, to expand our repertoire of disease biomarkers, therapeutic targets and druggability strategies, and elucidate mechanisms and versatility of anti-cancer drugs.

## Author contributions

VGD conceived and discussed the Research Topic. All the authors contributed to managing the published articles and reviewing them. All the authors contributed to the editorial manuscript writing and revision.
